# Antibiotic-induced gut microbiota dysbiosis has a functional impact on purine metabolism

**DOI:** 10.1186/s12866-023-02932-8

**Published:** 2023-07-13

**Authors:** Xin Liu, Leyong Ke, Ke Lei, Qian Yu, Wenqing Zhang, Changgui Li, Zibin Tian

**Affiliations:** 1 Department of Gastroenterology, The Affiliated Hospital of Qingdao University, No. 16 Jiangsu Road, Qingdao, 266000 China; 2 Department of Cosmetic surgery, Kunming Medical University, Kunming, 650000 China; 3 Center of Tumor Immunology and Cytotherapy, Medical Research Center, The Affiliated Hospital of Qingdao University, Qingdao, 266000 China; 4 Institute of Metabolic Diseases, Qingdao University, Qingdao, 266003 China

**Keywords:** Gut microbiota, Antibiotic, Purine metabolism, Purine salvage pathway

## Abstract

**Background:**

Dysbiosis of the gut microbiota is closely linked to hyperuricemia. However, the effect of the microbiome on uric acid (UA) metabolism remains unclear. This study aimed to explore the mechanisms through which microbiomes affect UA metabolism with the hypothesis that modifying the intestinal microbiota influences the development of hyperuricemia.

**Results:**

We proposed combining an antibiotic strategy with protein-protein interaction analysis to test this hypothesis. The data demonstrated that antibiotics altered the composition of gut microbiota as UA increased, and that the spectrum of the antibiotic was connected to the purine salvage pathway. The antibiotic-elevated UA concentration was dependent on the increase in microbiomes that code for the proteins involved in purine metabolism, and was paralleled by the depletion of bacteria-coding enzymes required for the purine salvage pathway. On the contrary, the microbiota with abundant purine salvage proteins decreased hyperuricemia. We also found that the antibiotic-increased microbiota coincided with a higher relative abundance of bacteria in hyperuricemia mice.

**Conclusions:**

An antibiotic strategy combined with the prediction of microbiome bacterial function presents a feasible method for defining the key bacteria involved in hyperuricemia. Our investigations discovered that the core microbiomes of hyperuricemia may be related to the gut microbiota that enriches purine metabolism related-proteins. However, the bacteria that enrich the purine salvage-proteins may be a probiotic for decreasing urate, and are more likely to be killed by antibiotics. Therefore, the purine salvage pathway may be a potential target for the treatment of both hyperuricemia and antibiotic resistance.

**Supplementary Information:**

The online version contains supplementary material available at 10.1186/s12866-023-02932-8.

## Background

The gut microbiota is composed of a large number of bacteria colonizing the gut and has been proven to widely affect inflammation [1], tumor growth [2], immunity [3], and metabolism [4]. Furthermore, within metabolism, gut microbiota can influence osteoporosis, which is mediated by amino acid metabolism [5], as well as type 2 diabetes, where microbiota dysbiosis can affect glucose metabolism and insulin sensitivity [6]. Purine metabolism is one of the major metabolic pathways [7]. Hyperuricemia (HUA) is defined as elevated levels of uric acid (UA) (> 6.8 mg/dL, serum UA) in the blood, and UA is the final product of purine metabolism [8]. There is a distinct difference in gut microbiota between HUA patients and healthy persons [9]. For example, enrichment of *Proteobacteria, Escherichia-Shigella genus* and decrease in the abundance of *Phascolarctobacterium, Bacteroides* and *Akkermansia* have been detected in HUA patients [10]. Moreover, *Acetobacter fabarum* possess genes that can encode xanthine dehydrogenase B (xdhB), urate hydroxylase A (urhA), and allantoinase E (puuE), which can utilize urate [11]. These results suggest that gut microbiota plays a nonnegligible role in the pathogenesis of HUA. However, the regulation of purine metabolism by the intestinal microbiota remains unclear.

Chemicals known as antibiotics have the ability to either kill or inhibit bacteria growth [12]. The widespread use of antibiotics has positively transformed the trajectory of infectious disease treatment and spread, at the expense of the gut microbiota [13]. Antibiotics have a broad and non-specific spectrum of action, affecting harmful and healthy bacteria simultaneously [14]. The impact of antibiotic treatment on gut microbiota has been widely investigated [15], with the overarching finding that it impairs the gut microbiota and GI epithelia [16]. Nevertheless, antibiotics have been shown to rebalance dysbiosis of the gut microbiota [12]. Numerous investigations have discovered that antibiotics’ modification of the gut microbiota results in clinical symptoms [17]. Based on this theory, Eiji Miyauchi et al. [18] found that mice administered antibiotics had a single bacterial strain depleted, resulting in attenuated symptoms of experimental autoimmune encephalomyelitis (EAE). This study provides a novel perspective on the relationship between gut microbiota and diseases. Given the important role of the gut microbiota in HUA, to our knowledge, no studies have been performed addressing the core microbiota of HUA by antibiotics, nor have any studies compared in vitro experimental results to demonstrate the mechanism of antibiotic-induced dysbiosis of the intestinal microbiota and purine metabolism.

In this study, we used an antibiotic strategy to investigate the interaction between the gut microbiome and UA metabolism. We found that the microbiome altered by antibiotics might perform a function linked to purine metabolism, especially in the salvage pathway of purine nucleotides.

## Results

### Antibiotic treatment disturbed the composition of gut microbiota

**Fig. 1:**
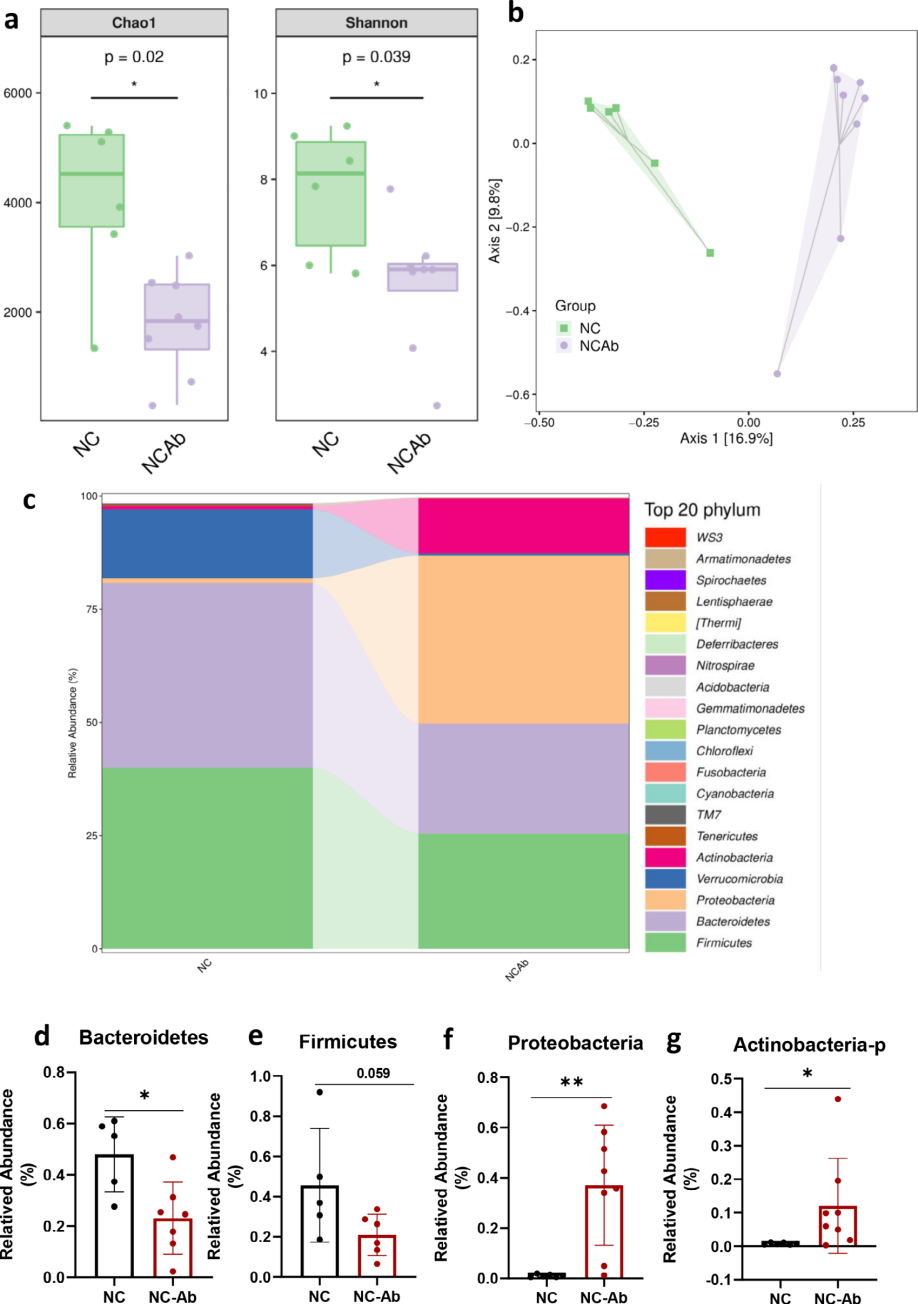
Antibiotic treatment disturbed the composition of gut microbiota. (**a**) Alpha-diversity among the NC-Ab and NC groups. (**b**) Unweighted UniFrac based PCoA analysis in the Ab-NC and NC groups. (**c**) The composition of microbiota in NC and NC-Ab groups, the top 20 phyla is shown. The relative abundance of (**d**) *Bacteroidetes*, (**e**) *Firmicutes*, (**f**) *Proteobacteria*, and (**g**) *Actinobacteria.* These results are expressed as mean ± SEM.*P < 0.05, **P < 0.01, vs. NC group. NC, control; NC-Ab, control-antibiotic-fed

To examine the role of the gut microbiota in UA metabolism, we subjected the C57BL/6J mice to an antibiotic cocktail (NC-Ab) or vehicle (NC) in their drinking water for 2 weeks. The 16 S rRNA gene sequencing analysis of the gut microbiota showed that the richness and diversity indices were significantly lower after antibiotic treatment (Chao [P = 0.02], Shannon [P = 0.039]) (Fig. 1a). Unweighted UniFrac-based principal coordinate analysis revealed that the microbiota of the NC-Ab group was easily distinguishable from that of the NC group (Fig. 1b). These results suggested that the composition of the microbiota was altered by antibiotic treatment. Moreover, at the phylum level, the relative abundances of *Bacteroidetes* and *Firmicutes* in NC-Ab mice were lower than those in NC mice, while the relative abundances of *Proteobacteria* and *Actinobacteria* in the NC-Ab group were higher than those in the NC group (Fig. 1c-g). At the genus level, the antibiotic-treated group was characterized by a higher richness of *Shigella, Enterococcus, Bifidobacterium, Bacteroides* and a lower abundance of *Lactobacillus* and *Allobaculum* (Fig. 5a).

### Antibiotic treatment elevated serum UA without liver and renal toxicity

**Fig. 2:**
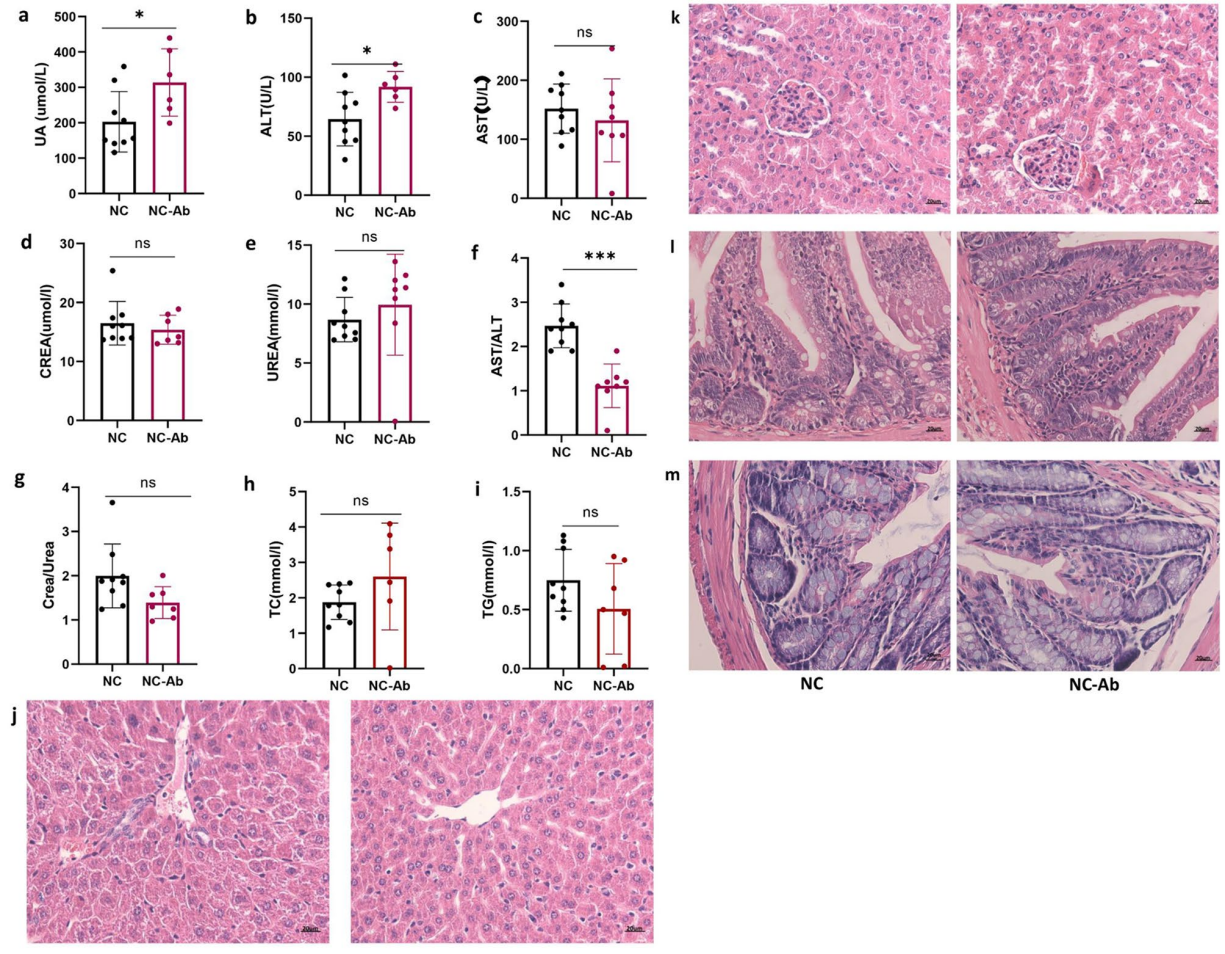
The effect of antibiotic on UA, liver, and renal function. (**a**) The serum UA levels after antibiotic treatment are shown. The concentration of ALT (**b**), AST (**c**), creatinine (**d**), urea (**e**), TC (**h**), and TG (**i**) in NC and NC-Ab groups. (**f**) The rate of AST and ALT. (**g**) The rate of creatinine and urea. Representative photomicrographs of H&E staining in mouse (**j**) liver, (**k**) kidney, (**l**) small intestine and, (**m**) colon tissues. These results are expressed as mean ± SEM.*P < 0.05, ***P < 0.001, vs. NC group. ALT, alanine aminotransferase; AST, aminotransferase; H&E, hematoxylin and eosin; NC, control; NC-Ab, control-antibiotic-fed; TC, total cholesterol; TG, triglyceride

The concentration of serum UA was 1.5 times greater in the NC-Ab group than the NC group after an antibiotic cocktail intervention for 2 weeks, suggesting that antibiotic management could regulate UA metabolism (Fig. 2a). We measured the effects of antibiotics on liver and renal function. There were no significant differences in aminotransferase (AST), creatinine, or urea levels between the groups, except for alanine aminotransferase (ALT) levels (Fig. 2b-g). Although serum ALT levels increased after antibiotic treatment, the AST-to-ALT ratio in the NC-Ab group was significantly decreased, rather than increased (Fig. 2f). ALT is primarily located in the cytoplasm of hepatocytes, and serves as a sensitive indicator of hepatocyte injury [19]. AST is predominantly distributed within liver cell mitochondria with only a small fraction present in the cytoplasm. Elevated levels of AST suggest more severe liver cell damage. Therefore, serum levels of both AST and ALT, as well as their ratio (AST/ALT), are commonly used to evaluate liver function [20]. The elevation of ALT levels alone is insufficient to fully characterize hepatic dysfunction, combining with the elevated AST and AST/ALT ratio to be clinically significant. Thus, the results suggested that antibiotic treatment elevated serum UA without liver toxicity. Furthermore, the creatinine-to-urea ratio was similar between the two groups (Fig. 2g). The liver is the primary organ involved in UA production whereas the kidneys and gut perform UA excretion. Tissues were observed microscopically after H&E staining. In the NC-Ab group, normal liver lobules, hepatocytes, and hepatic cords exhibited an orderly arrangement and were devoid of adipocytes (Fig. 2j). Moreover, serum triglyceride and total cholesterol levels did not increase after antibiotic treatment (Fig. 2h-i). Kidney sections prepared by H&E staining of NC-Ab mice displayed typical kidney architecture, regularly arranged cells, Bowman’s capsule, and renal tubules without dilatation (Fig. 2k). The small intestine and colon also had normal architecture. Intestinal villi and figure-like projections were intact without edema or shrinkage, and Paneth cells residing at the bottom of the crypts did not visibly vary between the two groups (Fig. 2l). Colon mucosa, in contrast to the small intestine, was not covered with villi; however, it contained deep tubular pits, additionally, no structural damage was discovered in the colon of antibiotic-treated mice (Fig. 2m). These data suggested that treatment with the antibiotic resulted in a sustained increase in serum UA levels without any evidence of liver or kidney toxicity.

### Antibiotic treatment altered the serum UA and gut microbiota in HUA model mice

**Fig. 3:**
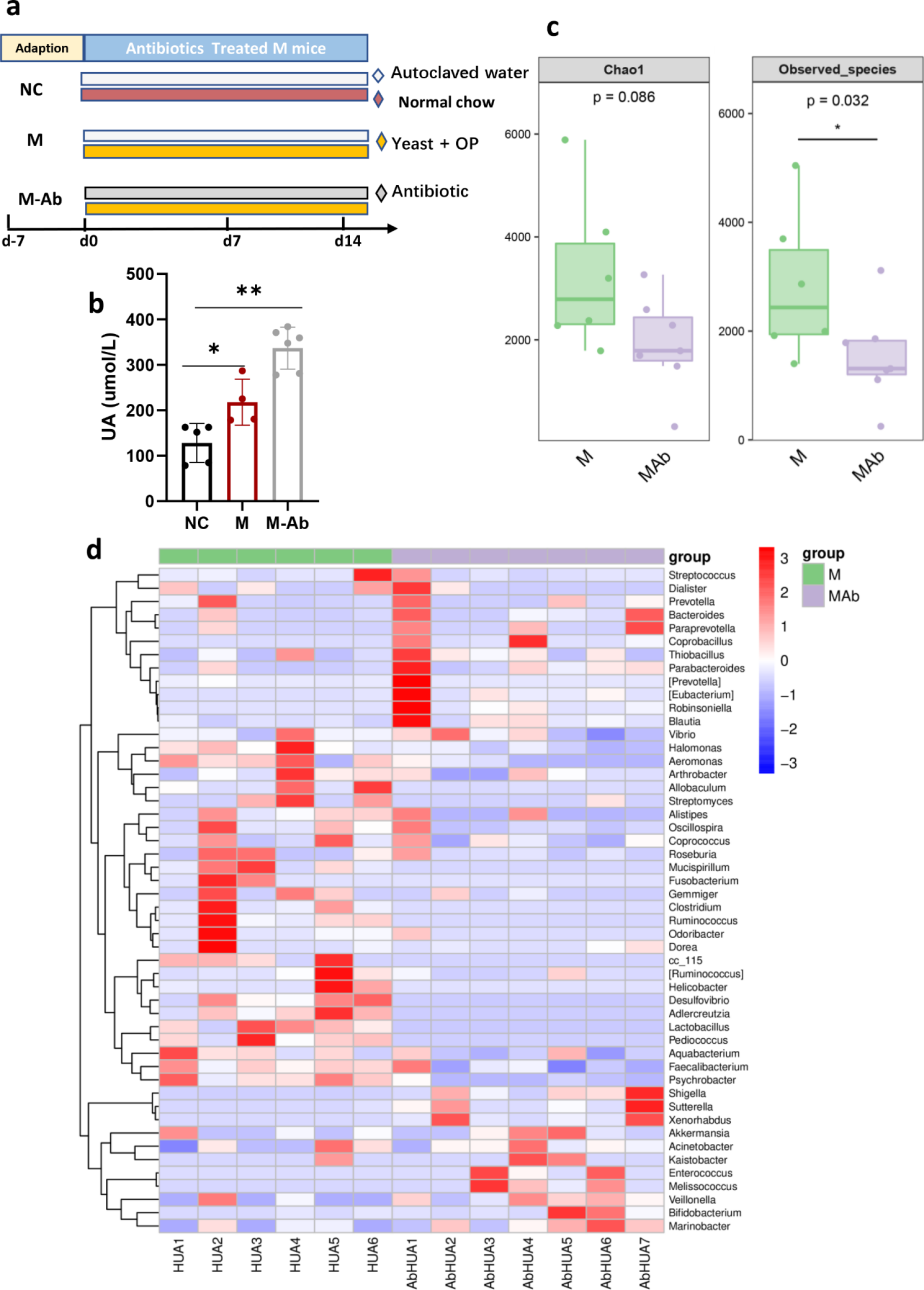
The effect of antibiotic on serum UA and gut microbiota in HUA model mouse. (**a**) A diagram illustrating the procedure of antibiotic treatment in HUA model mouse. C57BL/6J were randomly assigned to either NC group, hyperuricemia model group (M), or antibiotic-treated hyperuricemia model group (M-Ab). The NC and M groups were fed autoclaved water, while the M-Ab groups were fed the water containing an antibiotic cocktail (ampicillin 250 mg/L neomycin 250 mg/L and metronidazole 50 mg/L were mixed) and were fed the forage containing 5% yeast and 250 kg-1. d-1 potassium oxonate via peritoneal injection. After antibiotic treated for 14 days, all animals were sacrificed using CO2 inhalation. (**b)** Serum UA levels of groups were shown. (**c)** The Alpha-diversity among the M-Ab and M groups. **(d)** The heat map in the M-Ab and M groups. These results are expressed as mean ± SEM.*P < 0.05, **P < 0.01, vs. NC group. HUA, hyperuricemia; M, hyperuricemia model mouse; M-Ab, antibiotic-fed hyperuricemia model mouse; UA, uric acid; NC, control

Whether antibiotics regulate UA metabolism in hyperuricemic mice remains unclear. A mouse model of HUA was established using a high-yeast diet and per os intraperitoneal injection. The serum UA concentration in HUA model mice (M group) was obviously higher than those in the NC group, suggesting that the HUA model was successful (Fig. 3a-b). The mice were then administered an antibiotic cocktail (M-Ab) or vehicle (M) in their drinking water for 2 weeks. As Fig. 1b shows, the serum UA concentration of the M-Ab group was significantly increased after antibiotic management (Fig. 3b), which was consistent with the results of the antibiotic-treated normal UA mice (Fig. 2a), indicating that antibiotics can also elevate serum UA levels in HUA situation.Similarly, the richness and diversity indices were lower in the M-Ab group (Chao [P = 0.0086], observed species [P = 0.032]) than in the M group (Fig. 3c). Figure 3d shows that the composition of the microbiota differed between the M-Ab and M groups.

### Antibiotic intervention purine metabolism

**Fig. 4:**
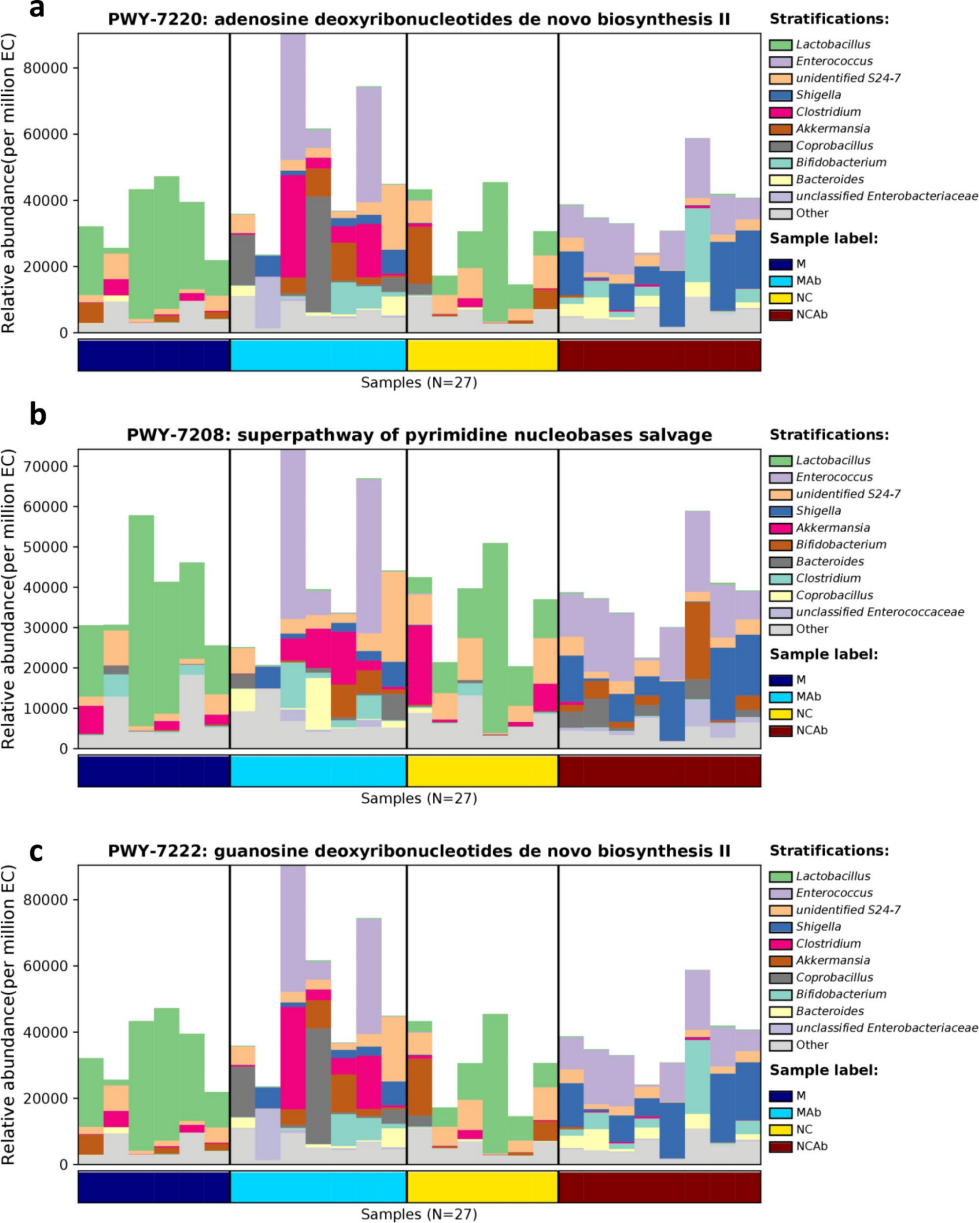
Predictive functional purine metabolism of microbial communities by PICRUSt2 algorithm. (**a**) Adenosine deoxyribonucleotides de novo biosynthesis. (**b**) Pyrimidine nucleobases salvage. (**c**) Guanosine deoxyribonucleotides de novo biosynthesis. PICRUSt2, phylogenetic investigation of communities by reconstruction of unobserved states

The 16 S rRNA gene profile is a crucial tool for studying microbial communities; however, it does not directly demonstrate the functional capability of a community. Alternatively, phylogenetic investigation of communities by reconstruction of unobserved states (PICRUSt) is a computer software that uses marker gene data and a library of reference genomes to predict the functional content of a metagenome. To determine gene families related to UA metabolism, the PICRUSt2 algorithm was employed using 16 S information. The results revealed that *Lactobacillus*, *Enterococcus, unidentified S24-7*, *Shigella*, *Clostridium*, *Akkermansia*, *Coprobacillus*, *Bifidobacterium*, and *Bacteroides* affected purine metabolism by participating in pyrimidine nucleobase salvage and de novo biosynthesis of adenosine and guanosine deoxyribonucleotides (Fig. 4a, b, c).

### Defining a key microbiota in the urate metabolism

**Fig. 5:**
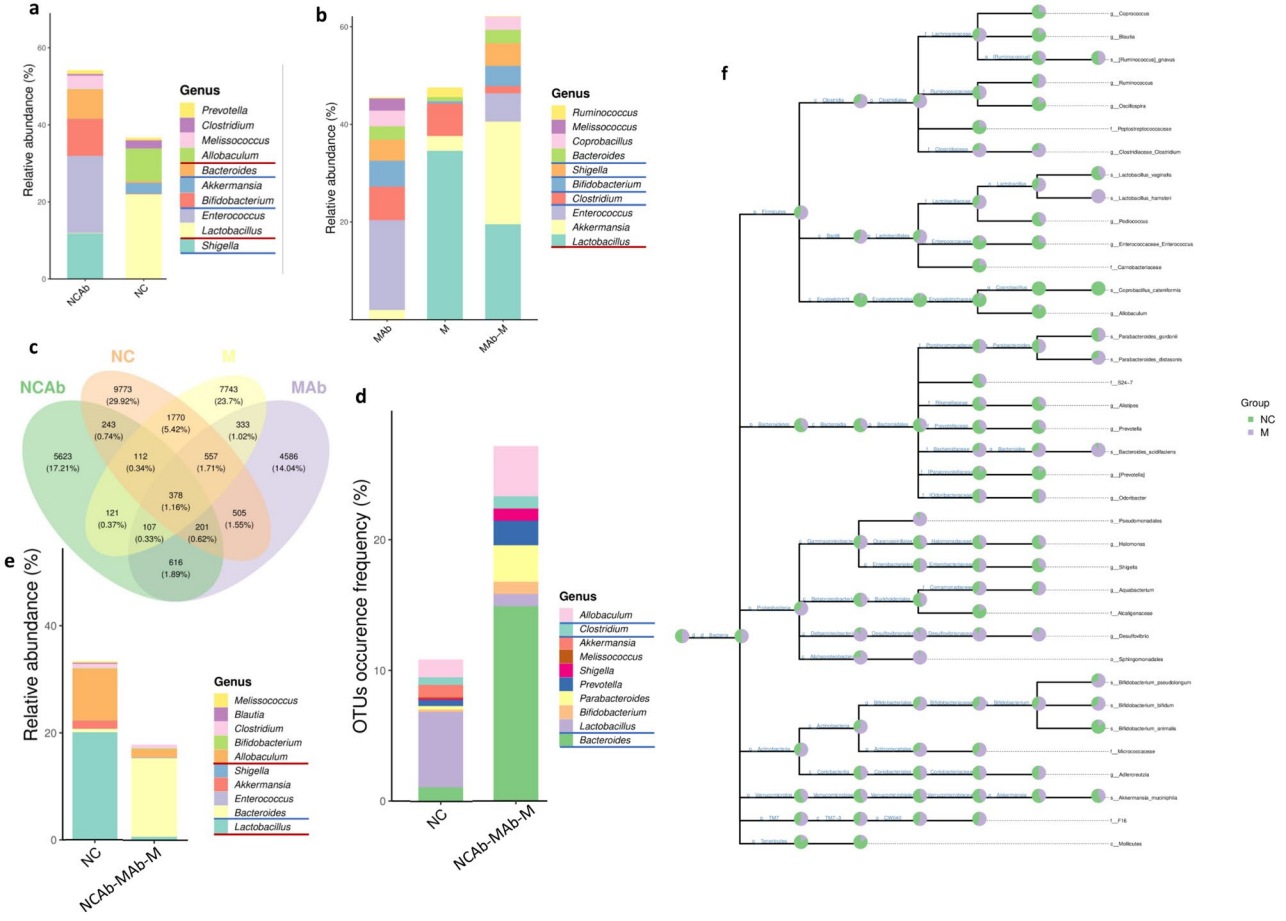
Defining a key microbiota in urate metabolism. (**a**) The relative abundance of genus in NC-Ab and NC groups. (**b**) The relative abundance of genus in M-Ab and M groups. (**c-d**) The Venn diagram of the four groups. (**e**) The relative abundance of bacteria at the genus level in NC and antibiotic treatment groups. (**f**) The taxonomic tree in NC and M groups. HUA, hyperuricemia; M, hyperuricemia model mouse; M-Ab, antibiotic-fed hyperuricemia model mouse; UA, uric acid; NC, control; NC-Ab, control-antibiotic-fed

Figure 5a–b shows the level of abundantly shared *Shigella*, *Enterococcus*, *Bifidobacterium*, *Bacteroides*, *Melissococcus* and *Lactobacillus* between both antibiotic groups at the genus level. Combining the results of the PICRUSt2 algorithm, *Shigella*, *Enterococcus*, *Bifidobacterium*, *Bacteroides*, and *Lactobacillus* may play a core role in the regulation of UA metabolism. Consistent with this result, we observed that the elevated UA groups (NC-Ab, HUA, and HUA-Ab) shared 107 overlapping ASVs. The shared ASVs at the genus level were *Bacteroides, Bifidobacterium, Prevotella*, and *Shigella* (Fig. 5c-d). As Fig. 5d displays, the most abundant ASVs/OTUs were found within the genus *Bacteroides*. *Lactobacillus* and *Allobaculum*, however, were lower in the high UA level groups (NC-Ab, M-Ab, and M) (Fig. 5e). Therefore, it appears that *Lactobacillus*, *Bacteroides*, and *Allobaculum* are important for UA metabolism. To clarify whether these microbiomes had the same tendency in HUA model mice, we compared the bacteria present in the antibiotic treatment and NC groups with the bacteria in the M and NC groups. As shown in Fig. 5f, *Bacteroides acidifacients*, *Bifidobacterium pseudolongum*, *Bifidobacterium bifidum*, *Ruminococus gnavus*, and *Colstridum* were in higher abundance and *Lactobacillus vaginalis* and *Allobaculum* were in lower abundance in M group. The STRING is a database devoted to protein-protein interaction (PPI) networks across the entire organism, and the network’s corresponding proteins can be identified enriched pathways [21]. We hence used the TRING database to explore a functional PPI network for the above-mentioned bacterial species. The results showed that *Bacteroides acidifacients* was significantly enriched (p = 1.32e-06) in one-carbon pool by folate, histidine metabolism, purine metabolism, biosynthesis of secondary metabolism, and metabolic pathways. *Additionally, Bifidobacterium bifidum* was similar to *Bacteroides* (p = 1.61e-07). *Bifidobacterium pseudolongum, Colstridum, Ruminococus gnavus, Allobaculum*, and *Lactobacillus vaginalis* were significantly enriched in purine metabolism and metabolic pathways (Table S1). These data suggest that key microorganisms are related to urate metabolism.

### A deficiency of the purine salvage pathway in the key bacteria of hyperuricemia

**Fig. 6:**
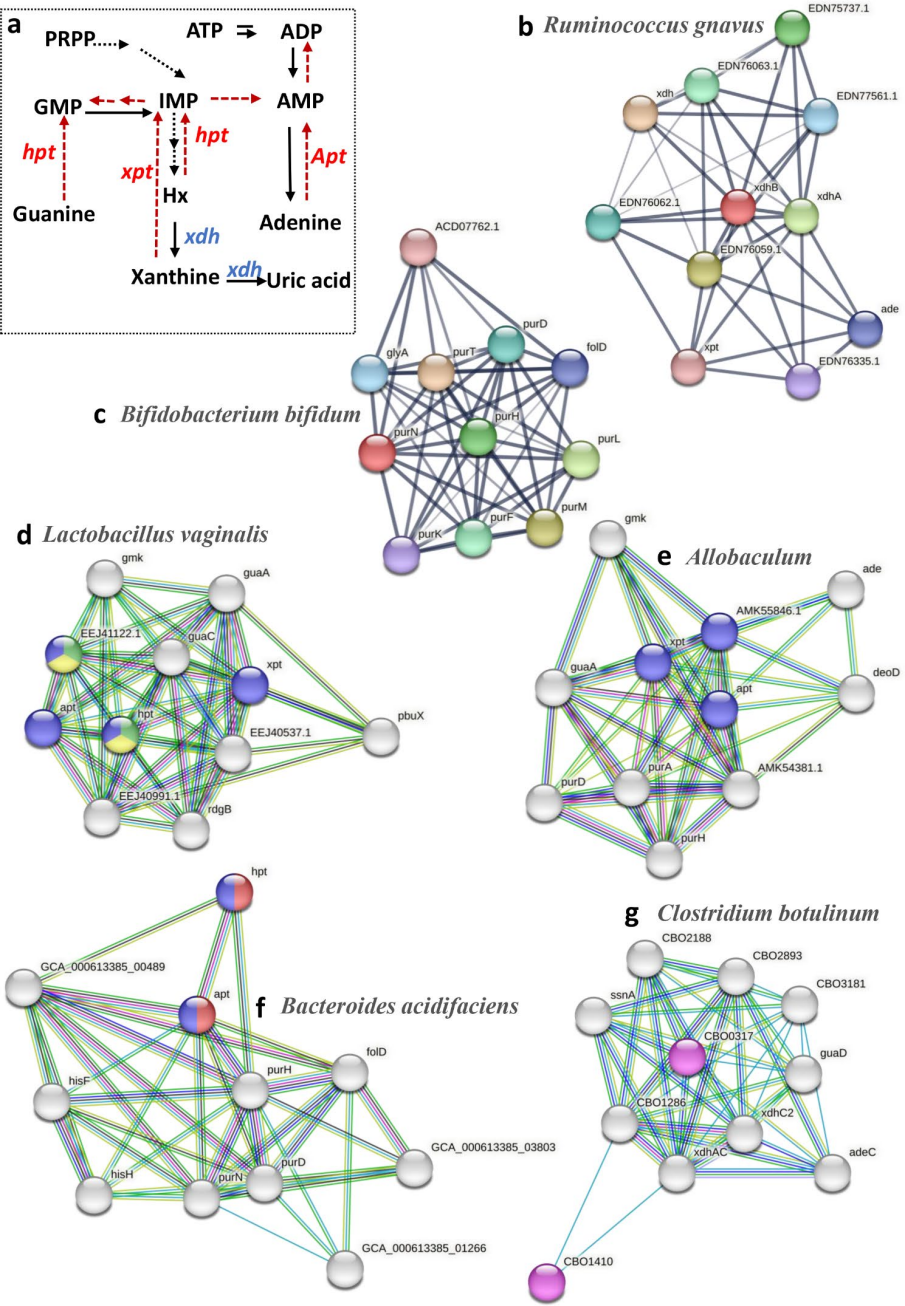
PPI analysis by the STRING. (**a**) The diagram of purine metabolism. Nodes were colored with specific role: Purine nucleotide salvage (red), purine ribonucleoside salvage (blue), IMP salvage (green), GMP salvage (yellow) and not purine salvage pathway (all colors). (**b**) *Ruminococcus gnavus* enriched the proteins of xhdB and xdhA without the proteins involved in salvage pathway. (**c**) *Bifidobacterium bifidum* enriched no the proteins of salvage pathway. (**d**) *Lactobacillus vaginalis* enriched in Purine nucleotide salvage (red), Purine ribonucleoside salvage, IMP salvage, GMP salvage and had four related proteins (**e)** *Allobaculum* enriched three proteins of salvage pathway. *Bacteroides acidifaciens* (**f**) and *Clostridium botulinum* (**g**) enriched two proteins of salvage pathway. xhd: xanthine dehydrogenase; apt: Adenine phosphoribosyltransferase; hpt: hypoxanthine phosphoribosyltransferase; pur: Phosphoribosylaminoimidazolecarboxamide formyltransferase

Purines are the main metabolites of nucleotide degradation, and they either degrade into UA or are utilized to synthesize nucleotides via a salvage pathway [22]. Enzymes are key factors that determine whether purines are reused to synthesize nucleotides or degraded to UA [23]. Hypoxanthine phosphoribosyltransferase (Hpt), Adenine phosphoribosyltransferase (apt), and xanthine phosphoribosyltransferase (xpt) are the enzymes crucial to the purine salvage pathway [24]. They reuse bases such as xanthine, hypoxanthine, adenine, and guanine to synthesize nucleotides (Fig. 6a). It has been reported that bacteria can utilize host purine metabolism by encoding purine salvage genes, and the UA level in hypoxanthine-guanine phosphoribosyltransferase deficiency patients is overproduced [25]. AS PPI network in the STRING can realize the underlying evidence each protein-protein association, enrich pathways and highlight proteins in the network [26], we used the PPI network to functionally predict bacterial purine metabolism. As Fig. 6f shows, seven proteins of *Bacteroides acidifaciens* were involved in purine metabolism (hpt, apt, Phosphoribosylaminoimidazolecarboxamide formyltransferase (pur) including purH, purD, purN, Methenyltetrahydrofolate cyclohydrolase (fold), and GCA-00489). The proteins involved in the salvage pathway of nucleotide synthesis, hpt and apt, were also enriched in *Bacteroides acidifaciens*. We next explored the functional protein association network focusing on the species of bacteria that were enriched in the M group (*Bifidobacterium pseudolongum*, *Bifidobacterium bifidum*, *Ruminococus gnavus*, and *Colstridum*). The results demonstrated that nine proteins overall from *Bifidobacterium bifidum* and *Ruminococus gnavus* were involved in the purine nucleotide biosynthetic process (de novo pathway), none of which were proteins in the salvage pathway (Fig. 6b-c). Moreover, the xanthine dehydrogenase (xdh) subunits xdhB and xdhA, were enriched in *Ruminococus gnavus* (Fig. 6b). Xdh is a core enzyme involved in UA synthesis, converting hypoxanthine and xanthine to UA to promote UA production (Fig. 6a). Additionally, in M group, enriched *Clostridium* and *Bifidobacterium pseudolongum*, were associated with two proteins involved in the salvage pathway (Fig. 6g and Figure S1a), similar to *Bacteroides acidifaciens*. Moreover, *Clostridium* was enriched in xdhs (xdhAC and xdhC2) (Fig. 6g). However, in the species enriched in control mice, four and three purine salvage-related proteins were found in *Lactobacillus vaginalis* (xpt, apt, hpt, and EEJ41122.1: hpt) and *Allobaculum* (xpt, apt, and AMK55846.1: hpt) (Fig. 6d-e). Hence, the data demonstrated that bacteria enriched in M group had two or fewer proteins related to the salvage pathway, lower than those enriched in the NC group. Moreover, *Lactobacillus vaginalis* and *Allobaculum* were enriched with four or three key enzymes of the salvage pathway, while the other species were only enriched in two key enzymes at most. The data suggest that the relative abundance of bacteria and enrichment of purine metabolism proteins were increased, and bacterial enrichment of salvage-related proteins was decreased in M group.

### Bacteria with sufficient quantities of purine salvage proteins decrease hyperuricemia

**Fig. 7:**
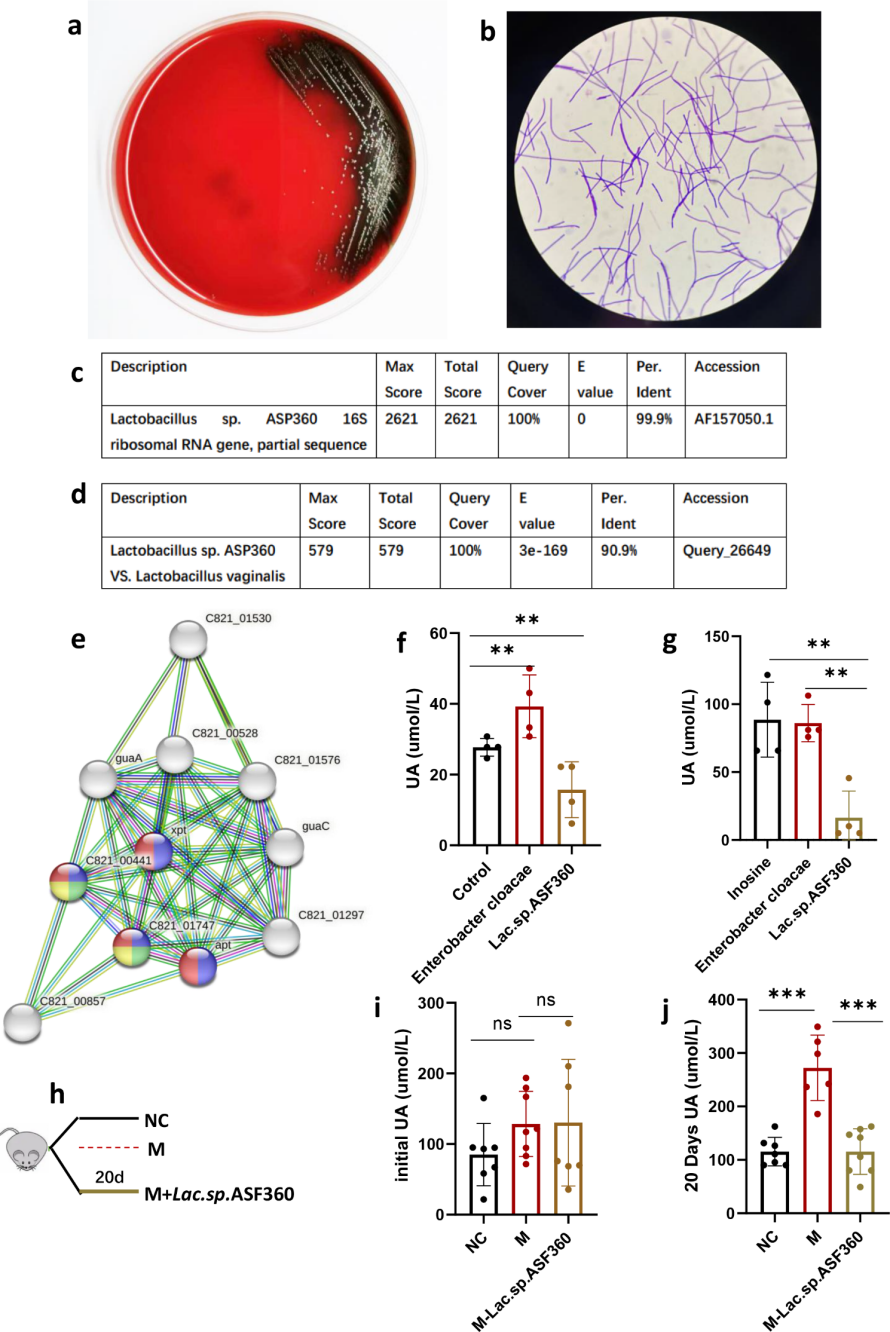
*Lactobacillus sp. ASF360* decreases hyperuricemia. (**a**) Morphology of *Lactobacillus sp. ASF360* on Columbia blood agar. (**b**) The morphology of *Lactobacillus sp. ASF360* under electron microscope. (**c**) *Lactobacillus sp. ASF360* was identified by 16 S rRNA gene sequences. (**d**) The sequence identity of *Lactobacillus sp. ASF360* and *Lactobacillus vaginalis* by BLAST. (**e**) The PPI analysis of *Lactobacillus sp. ASF360.* (**f**) The UA levels in the supernatant of *Lactobacillus sp. ASF360* and NCM-460 co-culture over 6 h. (**g**) The UA concentration in the supernatant of inosine treated *Lactobacillus sp. ASF360*. These results are expressed as mean ± SEM. **P < 0.01, vs. control group. (**h**) The diagram of *Lactobacillus sp. ASF360* intervention on the hyperuricemia mouse. (**i**) The initial UA before *Lactobacillus sp. ASF360* treatment. (**j**) The UA level after *Lactobacillus sp. ASF360* treatment for 20 days. These results are expressed as mean ± SEM.*P < 0.05, **P < 0.01, ***P < 0.001vs. M group. NC, control; M, hyperuricemia model mouse; M-lac.sp.ASF360, Lactobacillus sp. ASF360-fed hyperuricemia model mouse; XO, xanthine oxidase; UA, uric acid

*Lactobacillus vaginalis* had the greatest variety of salvage-related proteins and its effect on UA metabolism was examined. A *Lactobacillus* was isolated from mouse feces possessing a 16 S rRNA gene sequence. *Lactobacillus sp. ASF360* was the strain closest to the isolated *lactobacillus* (Fig. 7c). Gene sequences of *Lactobacillus sp. ASF360* with *Lactobacillus vaginalis* were assessed and the sequence identity between the two strains was found to be 90.9% (Fig. 7d). STRING analysis of *Lactobacillus sp. ASF360* revealed that four proteins involved in the salvage pathway (xpt, apt, C821-00441: Hpt, and C821-01747: Hpt) were significantly enriched, similar to *Lactobacillus vaginalis* (Fig. 7e). To the naked eye, colonies on Columbia blood agar were small, moist, light gray bumps with α or γ hemolytic rings (Fig. 7a). After Gram staining, the cells were Gram-positive bacteria with blue-purple in color. *Lactobacillus* cells exhibited a long-term threat (Fig. 7b). To examine the effect of *Lactobacillus sp. ASF360* on UA level, The culture experiments were conducted using intestinal epithelial cells NCM460 to assess the UA production (Fig. 7f), the data showed that NCM460 could produce UA in vitro, consistent with Wu’s research [27]. We then co-cultured *Lactobacillus sp. ASF360* and *Enterobacter cloacae* (positive control, which did not contain salvage-related proteins) with NCM-460 cells for 6 h, respectively (for the detailed experiment, refer to methods). The UA levels in the supernatant cultures of *Lactobacillus sp. ASF360* were lower than that in the negative and positive control groups (Fig. 7f). Next, we treated the live *Lactobacillus sp. ASF360* with inosine (1.25 mM) and found that the UA level in the inosine-treated group was significantly lower than that in the negative and positive control groups (Fig. 7g). These results indicate that *Lactobacillus sp. ASF360* decreased the UA level related to utilization of purines in vitro. In vivo, we referred to Eiji Miyauchi’s method [18], HUA model mice were treated with *Lactobacillus sp. ASF360* (M-Lac. sp.ASF360 group) for 20 days (Fig. 7h). The initial UA was no difference in the groups (Fig. 7i), after *Lactobacillus sp. ASF360* treated for 20 days, a reduction in serum UA levels in M-Lac.sp.ASF360 group was observed (Fig. 7j), suggesting that *Lactobacillus sp. ASF360* treatment could alleviate HUA. To examine whether the HUA was alleviated via changing host UA metabolism, particularly the liver, which is the largest UA-producing organ [28]. Firstly, we detected serum ALT and AST levels in all mice in the experiment. The results demonstrated that ALT, ALT and AST/ALT ratio did not change significantly after *Lactobacillus sp. ASF360* intervention (Figure S2a, b and c), suggesting that *Lactobacillus sp. ASF360* has no significant effect on the liver function. As xanthine oxidase (XO), catalyzing the conversion of hypoxanthine to xanthine and promoting xanthine to produce UA, is the key enzyme in the UA production [29], while other enzymes related to purine metabolism are not rate-limiting enzymes. Therefore, we then examined the hepatic XO concentration, the XO level of the liver was not significantly decreased after *Lactobacillus sp. ASF360* treatment, indicating that Lactobacillus sp. ASF360 did not inhibit UA production by inhibiting hepatic XO activity (Figure S2d). In addition to the liver, the kidney and small intestine also produce UA, we also tested the XO levels in the kidney and the intestine, the renal and intestinal XO levels also did not change after *Lactobacillus sp. ASF360* intervention (Figure S2e and Figure S2f). These results suggest that *Lactobacillus sp. ASF360* decreases serum UA levels in mice not by modulating host XO activity. We also isolated a *Bacteroides* strain (*Bacteroides fragilis*), which enriched in xpt and apt using STRING analysis (Figure S1b), and co-cultured them with NCM-460 cells for 6 h. The data demonstrated that the UA concentration in the supernatant did not differ between groups (Figure S1c). These results demonstrate that *Lactobacillus sp. ASF360* decreased UA levels both in vivo and in vitro, indicating the urate-lowering ability of *Lactobacillus sp. ASF360* is partly the result of the utilization of purines in the synthesis of nucleotides via the salvage pathway.

### The spectrum of antibiotics connected to the purine salvage pathway

**Fig. 8:**
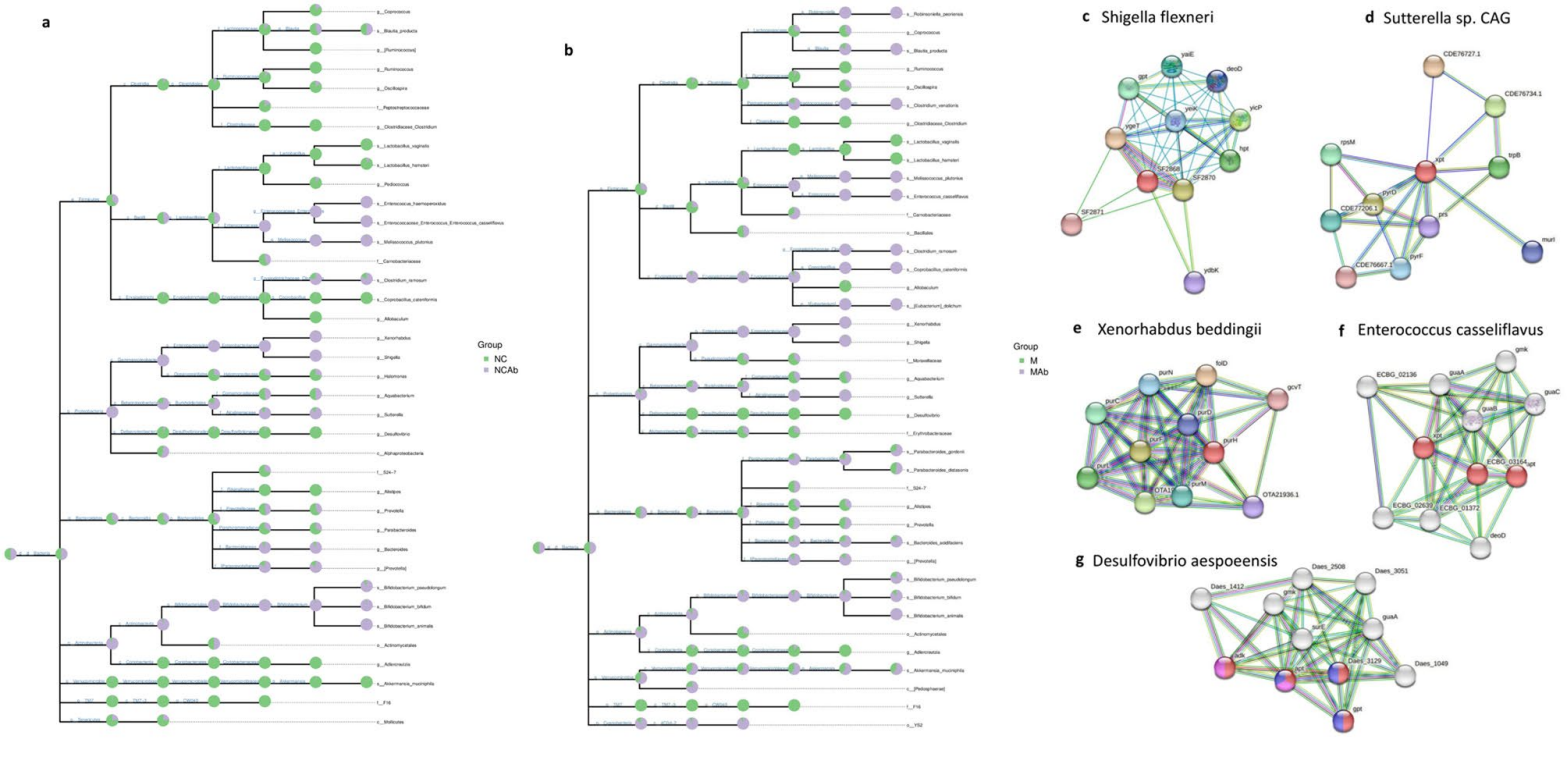
The antimicrobial spectrum was connected to the purine salvage pathway. (**a**) The taxonomic tree in NC and NC-Ab groups. (**b**) The taxonomic tree in M and M-Ab groups. The proteins involved in the salvage pathway from (**c**) *Shigella flexneri*, (**d**) *Sutterella*, (**e**) *Xenorhabdus* were absent. (**f**) *Enterococcus casseliflavus* enriched three of the proteins of the salvage pathway. (**g**) *Desulfovibrio aespoeensis* enriched four of the proteins of the salvage pathway. Nodes were colored with specific role: Purine nucleotide salvage (red), purine ribonucleoside salvage (blue), IMP salvage (green), GMP salvage (yellow) and not purine salvage pathway (all colors). HUA, hyperuricemia; M, hyperuricemia model mouse; M-Ab, antibiotic-fed hyperuricemia model mouse; UA, uric acid; NC, control; NC-Ab, control-antibiotic-fed

The phenomenon of antibiotics altering the composition of gut microbiota has been demonstrated. To elucidate the mechanism of this, we found that *Shigella flexneri, Sutterella, Enterococcus casseliflavus, Xenorhabdus, Bifidobacterium, Colstridum*, and *Bacteroides* were survivors, while *Desulfovibrio aespoeensis* and *Lactobacillus*, *Allobaculum* were depleted after antibiotic treatment (Fig. 8a-b). PPI analysis showed that there were no proteins involved in the salvage pathway from *Shigella flexneri*, *Xenorhabdus*, and *Sutterella*. *Bifidobacterium, Colstridum* (Fig. 8c-e), and *Bacteroides* had two or fewer proteins related to the salvage pathway (Fig. 6b, c, f). Interestingly, *Enterococcus casseliflavus* had three salvage-related proteins (Fig. 8f). However, four purine salvage-related proteins were found in *Desulfovibrio aespoeensis*, similar to *Lactobacillus vaginalis* (Fig. 8g). The results suggested that the antimicrobial spectrum of antibiotics may be connected to purine metabolism. The activation of the purine salvage pathway possibly accelerated the killing of the microbiome by antibiotics, while the bacteria lacking proteins associated with the salvage pathway likely escaped assassination by antibiotics.

## Discussion

Our research demonstrated that the core microorganisms of HUA model mice were the bacteria that enrich proteins involved in purine metabolism, with a higher abundance in elevated UA groups. The dysbiosis of gut microbiota have been discussed in previous studies [30], and the intestinal bacteria may be involved in the development of HUA [9]. However, the mechanism, and which microbiomes are key in HUA, remain unclear [31]. In the present study, we successfully elevated serum UA using antibiotic-induced intestinal microbiota disturbance; we also defined the core microbiota of HUA. We found that the antibiotic-induced microbiota dysbiosis was similar to that of the gut microbiota in M group, suggesting that the antibiotic strategy is a viable option to establish a direct link between gut microbiota and disease. Moreover, the relative abundance of *Lactobacillus* was reduced after antibiotic treatment and in M group, whereas *Bacteroides* was elevated. This data was similar to a cohort study in which *Bacteroides* was increased in the HUA patients [10]. Although there are no cohort reports that the abundance of *Lactobacillus* is reduced in HUA patients, numerous animal studies have demonstrated that *Lactobacillus* can decrease serum UA levels in HUA model animals [32].

In our work, we focused on the relationship between the UA level of HUA mice and the purine metabolism of the gut microbiota, and the results showed that the bacteria enriched in purine metabolism-associated proteins were enriched in the feces of HUA mice, while those enriched in proteins associated with the purine salvage pathway were significantly reduced. Previous studies on HUA focused on the purine metabolism [33], diet [34], and uric acid excretion of the host [35]. Little attention was paid to the purine metabolism of the gut microbiota itself, ignoring the role of the microbe’s purine metabolism in regulating the purine metabolism of the host. We observed that the bacteria enriching the purine salvage-proteins reduced UA both in vivo and in vitro, suggesting that gut microbes can use host purine metabolites to synthesize their own nucleotides, thereby decreasing the synthesis of UA in the host. However, numerous proteins and purine metabolites are involved in the purine salvage pathway, the expression of proteins and concentrations of metabolites, and the mechanism of them remains unclear, further study still is needed. The treatment of HUA is focused on promoting UA excretion, or decreasing the UA synthesis such as inhibiting xanthine oxidase in previous studies [35]. Our study suggested that supplementation with the bacteria enriching purine salvage-proteins may be a new target. It also seems that inhibition of those bacteria enriching proteins involved in purine metabolism may reduce UA level, but this hypothesis still needs to be proven in future studies. Our present study did not test this hypothesis, which is one of its shortcomings. However, it cannot be denied that this is a new entry point for the treatment of HUA. The purine metabolism in both the host and gut microorganisms is extremely complex [7], involving energy, nucleotide, and amino acid metabolism, as well as the interaction between the host and gut flora. Therefore, the underlying mechanisms need to be explored further. For example, which enzymes of purine metabolism are key enzymes to reduce host UA? Why are apt and hpt also expressed in *Bacteroides*, even though they do not reduce UA?

A novel finding in this study was that antibiotic-killed bacteria expressed more purine salvage pathway-related proteins, while the antibiotic-resistant bacteria had little or no purine salvage pathway-related proteins, suggesting that the purine salvage pathway may be relevant to the antibacterial spectrum of antibiotics. A recent study found that adenosine enhanced antibiotic killing tolerant bacteria were dependent on the purine salvage pathway [36]. The perspective of that study is bacterial resistance, while ours is the antibacterial spectrum, although the perspective is different, the findings coincide. Thus, the purine salvage pathway may provide new directions and possibilities for antibiotic-targeted therapy.

Given that purines play a central role in cellular physiology such as the synthesis of energy, as messengers in cell signaling, and nucleotide biosynthesis [37], Hitchings and Elion postulated that targeting specific biological processes in purine metabolic pathways might result in effective treatments [38]. This theory results in a series of drug designs and the subsequent development of drugs that act through purine biosynthetic pathways. For example, allopurinol, an inhibitor of xanthine oxidase, inhibits the synthesis of UA, and thus treats gout [39]. Azathioprine was designed as an anti-cancer drug based on the principle of disrupting purine metabolism and was later found to be more effective as an immunosuppressant [40]. Acyclovir is metabolized in vivo to produce purine analogs that terminate the synthesis of viral DNA [41]. Despite medical advances in targeting purine metabolism, many enzymes of this pathway remain unexplored, especially in the field of antibiotics [38]. Only a small number of researchers have begun to focus on purine metabolism and antibiotic resistance. The results demonstrated that bacterial metabolism is an important regulator of antimicrobial efficacy, and protect cells from antibiotic treatment via metabolic dormancy. They also revealed that the lethality of bactericidal antibiotics corresponds to the intracellular ATP concentration, and that bactericidal antibiotics increase central carbon metabolism activity [42]. Purine synthesis from de novo requires the involvement of carbohydrates [43], so it is not surprising that nucleotide metabolism plays a significant role in antibiotic effectiveness. Recent studies have found that 29% of all depleted genes in antibiotic persistence screens of *S. aureus* transposon mutant libraries were associated with cellular metabolism, five of which were involved in purine biosynthesis [44]. Moreover, purine nucleotides such as (p)ppGpp (the hyperphosphorylated nucleotides guanosine pentaphosphate and guanosine tetraphosphate) can induce antibiotic resistance by inhibiting cell proliferation and entering into the antibiotic persistence state [45]. (p)ppGpp can also directly inhibit enzymes (such as PurF and Gsk) in nucleotide metabolism [46]. These studies support the role of purine metabolism in the efficacy of antibiotic therapy. The newly identified purine salvage pathways may influence the antibacterial spectrum of antibiotics, although we have not further explored which purine metabolites or enzymes are key in influencing the antibacterial spectrum. For example, whether special enzymes of the purine salvage pathway influence their sensitivity of antibiotics via the (p)ppGpp pathway is still unknown, and needs to be verified in future studies. In addition, the present study only analyzed the number of enzymes related to the purine salvage pathway without further analyzing the specific mechanism. Therefore, we cannot yet fully explain why *Enterococcus* survived antibiotic treatment, suggesting that the antibiotic resistance genes of bacteria are non-negligible factors for antibiotic susceptibility and not just due to the purine salvage pathway alone (*Enterococcus* are known to be resistant to ampicillin, and their resistance mechanisms have been clearly described) [47].

The STRING is a database devoted to PPI networks across the entire organism and always used to analyze PPI of target genes [48], reveal the enrichment of PPI in gut microbiota [10] and identify the hub genes of purine metabolism [49]. We hence used The TRING database to explore the PPI of purine metabolism for bacterial species, the result that bacteria enriching purine salvage related proteins was less abundance in HUA mice and easy to be killed by antibiotic was a novel finding, although we could not completely rule out the possibility of inter-species PPI in our study, which is one of its shortcomings. Inter-species PPI is mainly focus on the interaction of host and pathogen [50] or host and parasite [51]. It appears unlikely that inter-species PPI affected the number of bacterial genes related to the purine salvage pathway (the target and result of our study). However, whether the inter-species PPI has the potential to influence the function of purine-related genes is still unclear, and further investigation is needed to understand the potential role and mechanisms involved.

Co-culture is an important method, especially co-culture with different cells is used to clarify the cellular interaction. As the important role of the intestinal microbiota, cell co-culture with bacteria has emerged in recent years, mainly for exploring the adhesion of intestinal microorganisms on intestinal epithelial cells, and gradually transitioning to explore the role of the microbiota on intestinal epithelial cells. For example, John O’Callaghan et al. exposed Caco-2 cells to Lactobacillus salvarius to understand how the strain modulates intestinal epithelial cell phenotypes [18]. Wu et al. co-cultured the supernatant of Lactobacillus with the NCM460 cells to understand the effect of Lactobacillus on the UA metabolism of cells [27]. In addition to co-culture of intestinal epithelial cells with bacteria, other cells such as soft tissue cells [52], human fibroblast [53], and osteoblast progenitor Cells [54] also were found co-cultured with bacteria. Moreover, co-culture of the intestinal epithelial cells with bacteria was also applied in humanized model systems to evaluate the treatment of drug [55], as well as metabolomics analysis of bacteria pathogen host cells in co-culture [56]. Thus, our choice of bacteria-cell co-culture method in our study to verify the bacteria rich in purine salvage related proteins whether decrease UA seems to be feasible. However, the mechanism how the strains regulate the UA metabolism and whether affect other metabolisms still needs further study.

## Conclusion

Our investigations demonstrated that antibiotics altered the composition of the gut microbiota, elevated the serum UA level, and regulated the purine metabolism of host as well as gut microbiota. The core microbiomes of HUA were related to the gut microbiota that enriched the purine metabolism related-proteins. And the bacteria enriching the purine salvage-proteins may be a probiotic for decreasing urate. An important consequence of this antibiotic-induced purine metabolic shift was that the antimicrobial spectrum of antibiotics was connected to the purine salvage pathway. Therefore, the purine salvage pathway may be a potential target for the treatment of HUA, as well as antibiotic resistance.

### Methods

#### Animals

A total of 42 male C57BL/6J mice aged 7 weeks were acquired from the Institute of Metabolic Diseases of Qingdao University (Qingdao, China). Standard food, water, and a chamber that was kept at a constant temperature of 23 °C and with 12 h/12 h light/dark cycle were given to the animals. The Affiliated Hospital of Qingdao University’s Animal Research Ethics Committee gave its approval to all animal research (No. AHQU-MAL20221012).

#### Antibiotic treatment

C57BL/6J at 7 weeks of age were randomly assigned to either the control (NC) group, antibiotic-fed control group (NC-Ab), hyperuricemia model group (M), or antibiotic-treated hyperuricemia model group (M-Ab). The NC and M groups were fed autoclaved water, while the NC-Ab and M-Ab groups were fed the water containing an antibiotic cocktail (ampicillin 250 mg/L neomycin 250 mg/L and metronidazole 50 mg/L were mixed). The M and M-Ab groups were fed the forage containing 5% yeast and 250 kg^− 1^. d^− 1^ potassium oxonate (PO, 97%; Sigma-Aldrich, St Louis, MO, USA) via peritoneal injection. The weight of the mice was tested every 2 days. Collect mouse feces, 24 h urine, and orbital blood every week. After 2 weeks, all animals were sacrificed. CO2 inhalation, the most common method of euthanasia for rodents [57], has the advantages of being low cost, relatively safe for the experimenters [58], and is acceptability when following the AVMA guidelines for the euthanasia of animals: 2020 edition[59]. The mice thus were euthanasia using CO2 inhalation. Briefly, to induce death as quickly and painlessly as possible, mouse was placed in an emptied, cleaned and transparent chamber and used compressed CO2 gas cylinders to introduced CO2 with a fill rate of 30-70% of the case volume/ min, maintaining CO2 flow for at least one minute after respiration ceases [59]. After confirmation of the death, the livers, kidneys, small intestines, and colons were carefully separated and weighed. Blood samples were centrifuged at 20 ± 5 °C for 2 h, and the resultant serum was collected. The samples were immediately frozen, and stored in − 80 °C refrigerators.

#### Hyperuricemia mouse model and bacteria ***Lactobacillus sp. ASF360*** colonization

The mice were randomly divided to the NC, M and M treated with *Lactobacillus sp. ASF360* (M-Lac.sp.ASP360) groups. The M-Lac.sp.ASP360 group was fed the water containing the antibiotic for 1 week to deplete the gut microbiota, before *Lactobacillus sp. ASF360* treatment, replaced the antibiotic-free water for 3 days [28]. The NC and M groups were given autoclaved water during this period. On the tenth day, the M and the M-Lac.sp.ASP360 groups received a peritoneal injection of 250 mg kg^− 1^. d^− 1^ PO and yeast-rich forage (5% yeast). The mouse of M-Lac.sp.ASP360 group received *Lactobacillus sp. ASF360* by gavage for 20 days (1 × 10^9^ colony forming units (UFC) /mouse) in 0.15 mL phosphate-buffered saline (PBS) (pH = 7.4). The NC group received only regular food and water, and intraperitoneal treatments with a PBS vehicle. The M group was given PBS by gavage. The serum UA concentration was tested once a week.

#### Histology analysis

The tissues including kidneys, small intestine, liver, as well as colon were washed in PBS to remove the blood and intestinal contents. The tissues were immediately fixed in a 4% paraformaldehyde solution, embedded in paraffin, separated into 5 μm sections, stained using hematoxylin and eosin (H&E), and observed with a microscope (200× magnification).

#### Gene sequencing and data analysis using 16 S ribosomal RNA

Fresh mouse feces were kept in − 80 °C refrigerators until DNA extraction. Total microbial DNA was extracted from frozen samples using QIAamp Fast DNA Stool Mini Kit (Qiagen, USA). After confirming the quality and concentration of DNA, for the pre-experiment a specific region of the 16 S rRNA gene was amplified via PCR using a specialized primer set. The following V3–V4 regions of the 16 S rRNA gene amplicon PCR primers were used: 5′-ACTCCTACGGGAGGCAGCA-3′ (forward primer) and 5′-GGACTACHVGGGTWTCTAAT-3′ (reverse primer). After purification, the PCR products were quantified using a Microplate Reader (BioTek Flx800). Sequencing libraries were prepared using TruSeq Nano DNA LT Library Prep Kit from Illumina (NEB, USA) according to the manufacturer’s recommendations. The samples were subjected to paired-end sequencing on an Illumina MiSeq/NovaSeq platform. Following this, sequencing analysis was performed using QIIME2 (2019,4), which can assess the alpha and beta diversities of samples based on the gglot2 pack. The different features of the bacteria in groups were identified using metagenomeSeq and LEfSe analyses.

#### Predictive function of microbial communities by phylogenetic investigation of communities by reconstruction of unobserved states (PICRUSt2)

Microbial functions were predicted using PICRUSt2 with MetaCyc (https://metacyc.org/) and KEGG (www.kegg.jp/kegg/kegg1.html) databases [60]. Species composition analysis of differential pathways was performed based on significantly differential metabolic pathways using a stratified sample metabolic pathway abundance table (path abun strat.tsv). Analysis steps: Call humanm2 barplot.py, set the relative abundance of bacteria as the vertical coordinate value, arrange the horizontal coordinate by sample group, and arrange the samples within the group by similarity; specify the specific metabolic pathway to be analyzed by “-f Spathway”. By default, we only analyzed the species composition of MetaCyc metabolic pathways with differences (analysis software: HUMAnN2). For subsequent comparative analysis, we defaulted to using parts per million of the summed abundance of KO and EC for each sample, respectively. The metabolic pathway abundance files (i.e., path_ abun_ unstrat.tsv and path_ abun_ strat. tsv) and the merit unit files (i.e., pred_ metagenome_ unstrat. tsv and pred_ metagenome_ strat. tsv) were normalized so that the abundance values were in units per million functional units. As a personalized option, we could also use the sum of species abundance (excluding species with NTSI > 2) for normalization. It should to be note that the sum of metabolic pathway abundances may not be equal across samples, but no further normalization is generally necessary.

#### PPI network

To identify the role of bacterial species shared between the high UA groups (NC-Ab, M, and M-Ab) in purine metabolism, The TRING database was used to build the PPI network (https://string-db.org). Statistical significance was set at p < 0.05.

#### Isolation and culture of ***Lactobacillus sp. ASF360*** and ***Enterobacter cloacae***

Fresh mouse feces were collected, mixed and tenfold serially diluted with sterile anaerobic PBS (pH 7.2, containing 0.1% L-cysteine). The 10^− 4^ dilutions were spread onto Colombian blood plates (Qingdao Hope Bio-Technology Co., Ltd., China;) and incubated at 37 °C under anaerobic conditions for 24 h. Colonies were selected according to bacterial colony morphology and α or γ hemolysis rings. All isolated strains were gram-stained, analyzed under a microscope, and identified using a Brooke MALDI-TOF mass spectrometer (IVD MALDI Bityper 2.3, Burker Scientific Technology Co., Ltd., China).

#### 16 S rRNA gene sequencing of ***Lactobacillus sp. ASF360***

High quality genomic DNA from *Lactobacillus sp. ASF360* was extracted using TIANamp Bacteria DNA Kit (TIANGEN BIOTECH (Beijing) CO., LTD). The 16 S rDNA was amplified according to the instruction manual. Identification of the isolated stains by BLAST based on the 16 S rDNA sequence. The morphology of *Lactobacillus sp. ASF360* cells were observed under an electron microscope (SEM, S-3400 N II Hitachi, Japan).

#### NCM460 cells co-culture with ***Lactobacillus sp. ASF360***

To measure UA levels in vitro, NCM460 cells (Shanghai Institute of Cell Biology, Chinese Academy of Sciences) were grown in 6-well tissue culture plates for 24 h. 12 and 2 h before adding the bacteria, the medium was replaced with antibiotic-free DMEM, respectively. After overnight culture, *Lactobacillus sp. ASF360* was collected, centrifuged, washed, and resuspended in PBS. The amount of the bacteria suspension was quantified using the optical density at 600 nm (OD600). Based on the equation of an OD600 of 1.0 equal to 2 × 10^8^ CFU/ml bacteria and an average of 2 × 10^6^ viable NCM640 cells per well within the 6-well plates, the number of bacteria added per well was calculated to satisfy a multiplicity of infection of 10:1, as previously reported [61]. After incubation for 6 h at 37 °C in the presence of 5% CO2, centrifugation at 12,000× g for 10 min, the supernatant was taken to detect using UA kit (Jiancheng, Nanjing, China).

#### ***Lactobacillus sp. ASF360*** treated with inosine

To measure the activity of *Lactobacillus sp. ASF360* degraded inosine, 10^9^*Lactobacillus sp. ASF360* cells were collected and treated with 1 mL trisodium phosphate solution (0.1 M) containing 1.25 mM inosine for 60 min. To prevent inosine further degradation, 0.1 ml of HClO4 (0.1 mL) was added to 0.9 mL of supernatant and the level of UA in the supernatant was measured by UA kits (Jiancheng, Nanjing, China).

## Electronic supplementary material

Below is the link to the electronic supplementary material.

Supplementary Material 1

Supplementary Material 2

## Data Availability

The datasets supporting the conclusions of this article are available in the NCBI Sequence Read Archive (SRA) repository under accession number PRJNA898992 (available on November 08 2022). Partial data generated or analyzed during the current study are available in the preprint server Research Square (https://www.researchsquare.com/article/rs-2261921/v1) with DOI: 10.21203/rs.3.rs-2261921/v1. And the rest data are included in this published article [and its supplementary information files].

## List of Abbreviations

HUA: Hyperuricemia
UA: Uric acid
PICRUSt2: Phylogenetic investigation of communities by reconstruction of unobserved states
PPI: Protein–protein interaction
ASVs/OTUs: Variants/operational taxonomic units
ALT: Alanine aminotransferase
AST: Aminotransferase
TC: Total cholesterol
TG: Triglyceride
xhd: Xanthine dehydrogenase
apt: Adenine phosphoribosyltransferase
hpt: Hypoxanthine phosphoribosyltransferase
pur: Phosphoribosylaminoimidazolecarboxamide formyltransferase
