# Supplementary material for: Antibiotic-induced gut microbiota dysbiosis has a functional impact on purine metabolism
Source: BMC Microbiol. 2023 Jul 13;23:187. doi: 10.1186/s12866-023-02932-8 (PMC10339580; doi:10.1186/s12866-023-02932-8)
Supplement: Supplementary file 1 — Supplementary Material 1 [file 12866_2023_2932_MOESM1_ESM.docx]

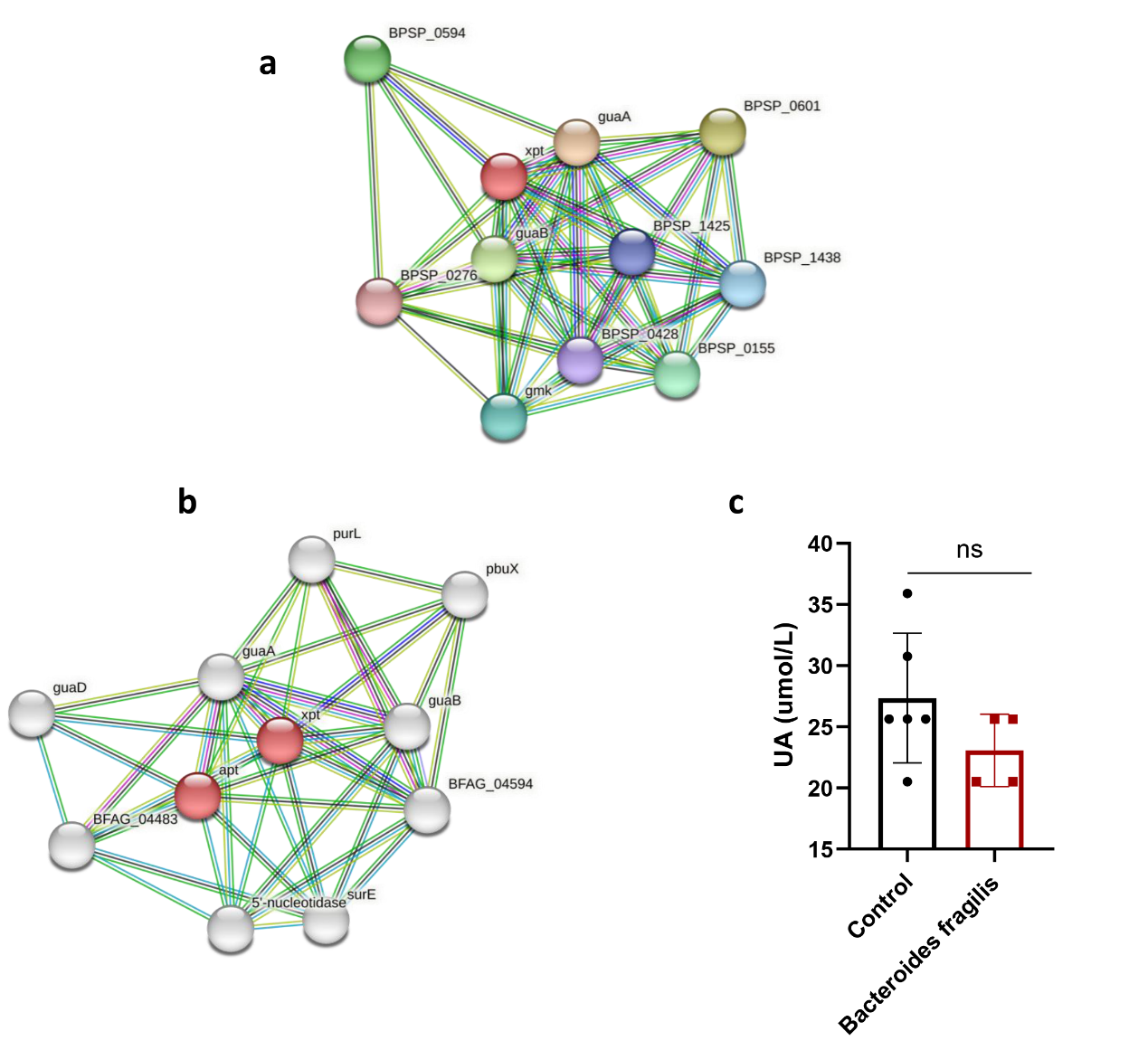


**Figure S1** PPI analysis by the STRING and the effect of *Bacteroides fragilis* on NCM460 cells. **(a)** The PPI analysis of *Bifidobacterium pseudolongum* by the STRING. (**b**) The PPI analysis of *Bacteroides fragilis* by the STRING. (**c**) The UA levels in the supernatant of *Bacteroides fragilis* and NCM-460 co-culture over 6 h.


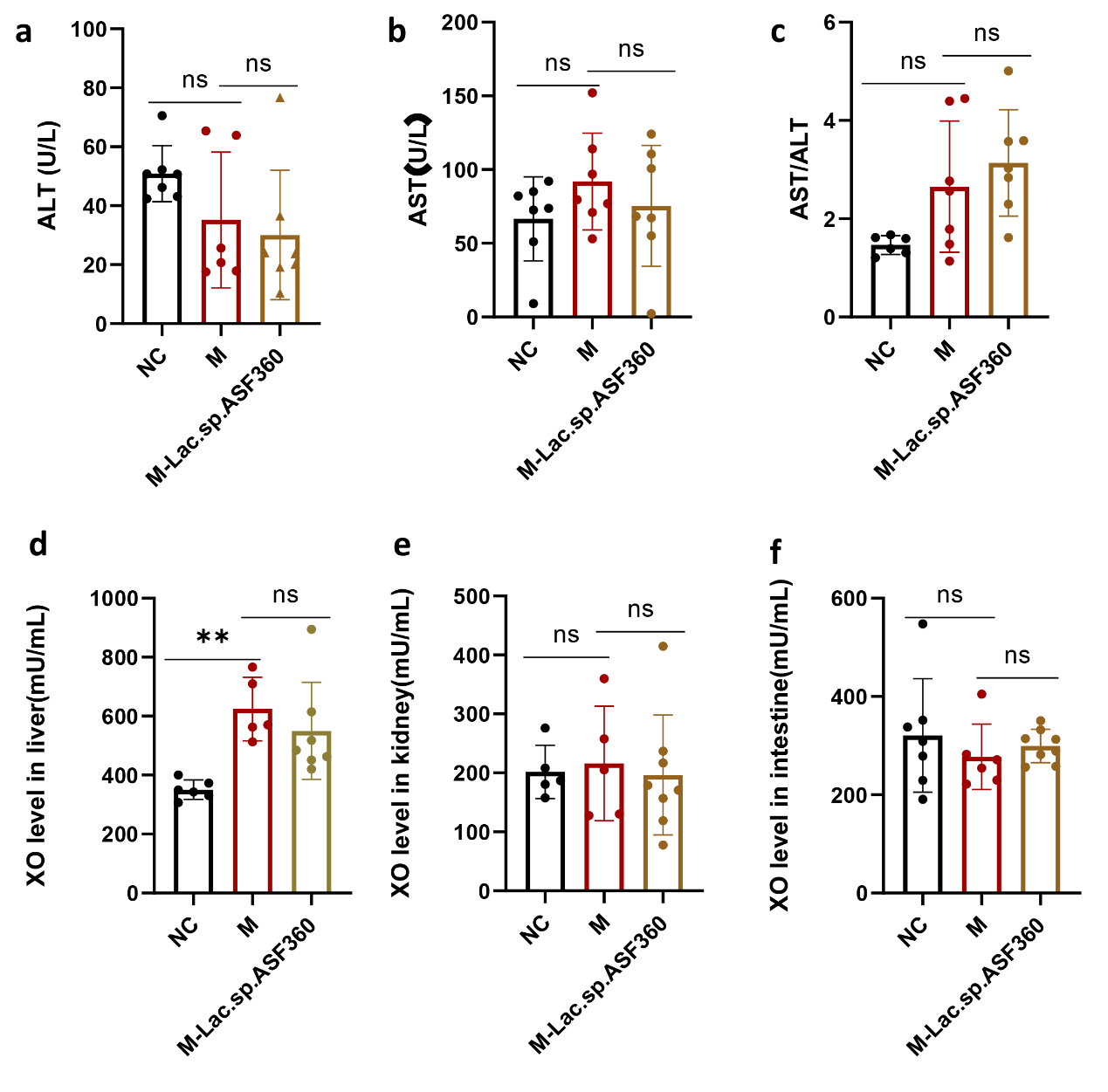


**Figure S2** The effect of Lactobacillus sp. ASF360 on liver function and XO activity. The concentration of ALT (**a**), AST (**b**), and AST/ALT ratio (**c**) in three groups. The XO level of liver (**d**), kidney (**e**) and intestine (**f**). These results are expressed as mean ± SEM.*P < 0.05, **P < 0.01, vs. NC group. ALT, alanine aminotransferase; AST, aminotransferase; XO, xanthine oxidase; NC, control; M, hyperuricemia model mouse; M-lac.sp.ASF360, Lactobacillus sp. ASF360-fed hyperuricemia model mouse
