# Supplementary material for: Antibiotic-induced gut microbiota dysbiosis has a functional impact on purine metabolism
Source: BMC Microbiol. 2023 Jul 13;23:187. doi: 10.1186/s12866-023-02932-8 (PMC10339580; doi:10.1186/s12866-023-02932-8)
Supplement: Supplementary file 2 — Supplementary Material 2 [file 12866_2023_2932_MOESM2_ESM.docx]

**Table S1** KEGG pathways by STRING analysis (www.kegg.jp/kegg/kegg1.html)

| Bacteria | Pathways | Description | Count in network | strength | False discovery rate |
| --- | --- | --- | --- | --- | --- |
| *Bacteroides acidifaciens* | map00670 | One carbon pool by folate | 5 of 25 | 1.95 | 2.84e-07 |
|  | [map00340](https://www.kegg.jp/kegg-bin/show_pathway?map00340) | Histidine metabolism | 2 of 19 | 1.67 | 0.0198 |
|  | map00230 | Purine metabolism | 6 of 134 | 1.3 | 1.04e-05 |
|  | map01100 | Metabolic pathways | 11 of 1349 | 0.56 | 2.47e-05 |
|  | map01110 | Biosynthesis of secondary metabolites | 7 of 456 | 0.84 | 0.00039 |
| *Bifidobacterium bifidum* | map00670 | One carbon pool by folate | 4 of 14 | 1.69 | 8.90e-05 |
|  | map00230 | Purine metabolism | 9 of 84 | 1.57 | 1.04e-10 |
|  | map01110 | Biosynthesis of secondary metabolites | 8 of 230 | 0.78 | 0.00016 |
|  | map01100 | Metabolic pathways | 11 of 595 | 0.5 | 0.00011 |
| *Bifidobacterium pseudolongum* | map00230 | Purine metabolism | 10 of 52 | 1.42 | 7.06e-12 |
|  | map01100 | Metabolic pathways | 10 of 545 | 0.4 | 0.0160 |
| *Clostridium botulinum* | map00230 | Purine metabolism | 6 of 80 | 1.39 | 7.56e-06 |
|  | map01100 | Metabolic pathways | 11 of 595 | 0.53 | 8.40e-05 |
| *Ruminococcus gnavus* | map00230 | Purine metabolism | 7 of 48 | 2.38 | 2.13e-09 |
|  | map01100 | Metabolic pathways | 7of 537 | 0.67 | 0.0089 |
| *Allobaculum* | fro00230 | Purine metabolism | 8of 52 | 1.59 | 4.22e-10 |
|  | fro01100 | Metabolic pathways | 10 of 714 | 0.55 | 0.00052 |
| *Lactobacillus vaginalis* | map01100 | Metabolic pathways | 7of 537 | 0.67 | 0.0089 |
|  | map00230 | Purine metabolism | 10 of 48 | 1.56 | 2.53e-13 |
